# Stringent comparative sequence analysis reveals SOX10 as a putative inhibitor of glial cell differentiation

**DOI:** 10.1186/s12864-016-3167-3

**Published:** 2016-11-07

**Authors:** Chetna Gopinath, William D. Law, José F. Rodríguez-Molina, Arjun B. Prasad, Lingyun Song, Gregory E. Crawford, James C. Mullikin, John Svaren, Anthony Antonellis

**Affiliations:** 1 Cellular and Molecular Biology Program, University of Michigan Medical School, Ann Arbor, MI 48109 USA; 2 Department of Human Genetics, University of Michigan Medical School, Ann Arbor, MI 48109 USA; 3 Cellular and Molecular Pathology Program, University of Wisconsin-Madison, Madison, WI 53706 USA; 4 Genome Technology Branch, National Human Genome Research Institute, National Institutes of Health, Bethesda, MD 20892 USA; 5 Center for Genomic and Computational Biology, Duke University Medical Center, Durham, NC 27708 USA; 6 Department of Pediatrics, Duke University Medical Center, Durham, NC 27708 USA; 7 Waisman Center, University of Wisconsin-Madison, Madison, WI 53706 USA; 8 Department of Comparative Biosciences, University of Wisconsin-Madison, Madison, WI 53706 USA; 9 Department of Neurology, University of Michigan Medical School, Ann Arbor, MI 48109 USA

**Keywords:** Comparative sequence analysis, Transcriptional regulation, Ultra-conserved sequences, Myelination, Schwann cells, SOX10

## Abstract

**Background:**

The transcription factor SOX10 is essential for all stages of Schwann cell development including myelination. SOX10 cooperates with other transcription factors to activate the expression of key myelin genes in Schwann cells and is therefore a context-dependent, pro-myelination transcription factor. As such, the identification of genes regulated by SOX10 will provide insight into Schwann cell biology and related diseases. While genome-wide studies have successfully revealed SOX10 target genes, these efforts mainly focused on myelinating stages of Schwann cell development. We propose that less-biased approaches will reveal novel functions of SOX10 outside of myelination.

**Results:**

We developed a stringent, computational-based screen for genome-wide identification of SOX10 response elements. Experimental validation of a pilot set of predicted binding sites in multiple systems revealed that SOX10 directly regulates a previously unreported alternative promoter at *SOX6*, which encodes a transcription factor that inhibits glial cell differentiation. We further explored the utility of our computational approach by combining it with DNase-seq analysis in cultured Schwann cells and previously published SOX10 ChIP-seq data from rat sciatic nerve. Remarkably, this analysis enriched for genomic segments that map to loci involved in the negative regulation of gliogenesis including *SOX5*, *SOX6*, *NOTCH1*, *HMGA2*, *HES1*, *MYCN*, *ID4*, and *ID2*. Functional studies in Schwann cells revealed that: (1) all eight loci are expressed prior to myelination and down-regulated subsequent to myelination; (2) seven of the eight loci harbor validated SOX10 binding sites; and (3) seven of the eight loci are down-regulated upon repressing SOX10 function.

**Conclusions:**

Our computational strategy revealed a putative novel function for SOX10 in Schwann cells, which suggests a model where SOX10 activates the expression of genes that inhibit myelination during non-myelinating stages of Schwann cell development. Importantly, the computational and functional datasets we present here will be valuable for the study of transcriptional regulation, SOX protein function, and glial cell biology.

**Electronic supplementary material:**

The online version of this article (doi:10.1186/s12864-016-3167-3) contains supplementary material, which is available to authorized users.

## Background

Schwann cells produce the myelin sheath in the peripheral nervous system (PNS), which allows rapid saltatory conduction and long-range communication between the central nervous system and innervated muscles and sensory organs. Schwann cell development is directed by a transcriptional hierarchy that promotes the expression of proteins important for migration along peripheral nerves, radial sorting of axons, and the initiation of myelination [1, 2]. Atop this hierarchy sits the transcription factor SOX10, which is critical for the development and long-term function of Schwann cells [3] and is expressed during all stages of Schwann cell development [3, 4].

Three major lines of evidence underscore the importance of SOX10 for the function of Schwann cells. First, ablation of *Sox10* expression in mouse models causes: (*i*) a lack of Schwann cells when performed during early development [4]; (*ii*) lethality due to peripheral neuropathy when performed in immature Schwann cells [5]; and (*iii*) demyelination of peripheral nerves when performed in terminally differentiated Schwann cells [6]. Second, dominant-negative *SOX10* mutations cause an autosomal dominant disease characterized by peripheral demyelinating neuropathy, central dysmyelinating leukodystrophy, Waardenburg-Shah syndrome, and Hirschsprung disease [7, 8]; the non-PNS phenotypes reflect the role of SOX10 in other neural crest derivatives (*i.e.*, melanocytes and enteric neurons) and in oligodendrocytes. Finally, mutations in SOX10 target genes including those encoding peripheral myelin protein 22 (*PMP22*), myelin protein zero (*MPZ*), and gap junction beta 1 (*GJB1*) cause demyelinating peripheral neuropathy [9–13].

The identification of additional SOX10 response elements and target loci will provide important information on the process of myelination in the peripheral nerve as well as novel target sequences to scrutinize for mutations and modifiers of peripheral neuropathy. Indeed, genome-wide analyses have been essential for characterizing SOX10 biology in Schwann cells [14, 15]; however, these efforts have primarily focused on identifying positive regulators of myelination by examining tissues or cells in a myelinating state. Thus, less-biased approaches are needed to complement the above studies and to identify functions of SOX10 outside of the regulation of promyelinating loci.

Here, we describe a stringent computational strategy to rapidly predict SOX10 response elements in the human genome. Combined with molecular functional studies, this strategy revealed that SOX10 positively regulates the expression of *SOX5*, *SOX6*, *NOTCH1*, *HMGA2*, *HES1*, *MYCN*, *ID4*, and *ID2*. Interestingly, each of these genes has a known or predicted role in the negative regulation of glial cell differentiation. As such, we identified a putative novel role for SOX10 in Schwann cells and present a model where SOX10 activates the expression of negative regulators of myelination to temper the pro-myelinating program during non-myelinating stages of Schwann cell development.

## Results

### Genome-wide prediction of SOX10-responsive transcriptional regulatory elements

SOX10 binds to a well-defined consensus sequence (‘ACACA’ or ‘ACAAD’; where ‘D’ is a G, T, or A nucleotide) as a monomer or as a dimer when two consensus sequences are oriented in a head-to-head fashion [14, 16]. To identify putative SOX10 binding sites in the human genome, we wrote a Perl script to scan each human chromosome and report all occurrences of the above SOX10 consensus sequence. This revealed over 33 million monomeric consensus sequences (Additional file 1) and ~549,000 dimeric consensus sequences with an intervening sequence of five to 10 base pairs (Additional file 2).

Multiple-species conservation analysis is an effective approach for predicting non-coding DNA sequences with a role in transcriptional regulation [17]. Importantly, functionally validated SOX10 binding sites have been identified in non-coding genomic sequences that are conserved between human and chicken [18–20]. To prioritize the large dataset of SOX10 consensus sequences, we aligned the human, mouse, and chicken genomes and identified all genomic sequences that are five base pairs or longer (the length of the monomeric SOX10 consensus sequence) and that are identical between these three species. This revealed over two million conserved coding and non-coding genomic segments (Additional file 3).

To develop a panel of prioritized SOX10 consensus sequences for functional studies, we used the rationale that: (1) focusing on conserved dimeric SOX10 consensus sequences will enrich for *bona fide* SOX10 binding sites; (2) focusing on non-coding sequences will deprioritize sequences that are conserved due to the function of the gene product; and (3) focusing on proximal promoter and intronic sequences will provide a candidate target gene for further studies. Thus, we compared the above datasets to identify dimeric SOX10 consensus sequences that are conserved between human, mouse, and chicken (including the intervening sequence), reside in non-coding sequences, and map to an intron or 2.5 kb upstream or downstream of a known (RefSeq) human gene. This revealed 238 genomic sequences at 160 loci for further study (Additional file 4). To determine the efficacy of our approach, we further prioritized the above 238 genomic segments by identifying the subset that map to loci with a known or predicted role in myelination (see methods for details). This revealed 57 genomic sequences at 32 loci with a conserved, dimeric SOX10 consensus sequence that resides within an intron or directly upstream of a myelin-related transcriptional unit; we named these elements SOX10 Conserved Consensus Sequences (SOX10-CCS; Additional file 5).

### Seven conserved SOX10 consensus sequences display regulatory activity in Schwann cells

**Fig. 1:**
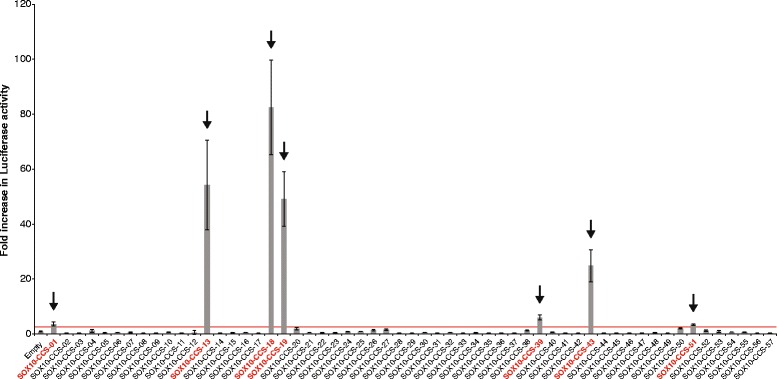
Seven regions demonstrate regulatory activity in Schwann cells. Each of the 57 genomic segments containing prioritized SOX10 consensus sequences was cloned upstream of a luciferase reporter gene and tested for enhancer activity in cultured Schwann (S16) cells. Luciferase data are expressed relative to a control vector that does not harbor a genomic insert (‘Empty’). Regions that display a greater than 2.5-fold increase (red line) in luciferase activity are indicated in red text and by an arrow. Error bars indicate standard deviations

Using our computational pipeline, we identified 57 regions that harbor conserved head-to-head SOX10 consensus sequences at loci with a known or predicted role in myelination. To test if these sequences are active in Schwann cells in vitro, a region surrounding each consensus sequence (Additional file 5) was amplified from human genomic DNA and cloned upstream of a minimal promoter directing the expression of a luciferase reporter gene. The regulatory activity of each genomic segment was tested in cultured rat Schwann (S16) cells [21, 22], which express endogenous SOX10 [19]. The luciferase expression directed by each genomic segment was determined in luciferase activity assays compared to a control vector with no genomic insert (‘Empty’). Seven of the 57 genomic segments demonstrated a greater than 2.5-fold increase in luciferase activity compared to the empty vector in S16 cells (Fig. 1): SOX10-CCS-01 (3.7-fold increase; maps to *PAX7*), SOX10-CCS-13 (54-fold increase; maps to *SOX6*), SOX10-CCS-18 (82-fold increase; maps to *SOX5*), SOX10-CCS-19 (49-fold increase; maps to *SOX5*), SOX10-CCS-39 (5.9-fold increase; maps to *TCF7L2*), SOX10-CCS-43 (25-fold increase; maps to *BCAS3*), and SOX10-CCS-51 (2.6-fold increase; maps to *NFIB*). These data suggest that these seven genomic sequences (Table 1) are potential SOX10 response elements.

**Table 1:** Seven genomic segments with regulatory activity in Schwann cells

Element ID	Locus	UCSC coordinates^a^	SOX10 Consensus sequence^b^
SOX10-CCS-01	*PAX7*	chr1:18,854,300-18,855,055	**ACAAA**CTCATTAAA**CTTGT**
SOX10-CCS-13	*SOX6*	chr11:16,334,301-16,335,278	**ACAAT**CAAGC**ATTGT**
SOX10-CCS-18	*SOX5*	chr12:24,058,988-24,059,872	**ACAAA**AATGT**ATTGT**
SOX10-CCS-19	*SOX5*	chr12:24,059,397-24,060,164	**ACACA**GAACATT**ATTGT**
SOX10-CCS-39	*TCF7L2*	chr10:114,894,980-114,895,808	**ACAAT**CCCCAAGATT**TTTGT**
SOX10-CCS-43	*BCAS3*	chr17:56,683,905-56,684,657	**ACACA**TTAATAACGT**TTTGT**
SOX10-CCS-51	*NFIB*	chr9:14,299,332-14,299,796	**ACAAT**CTGTTCTT**TGTGT**

^a^Coordinates refer to the March 2006 UCSC Genome Browser Human assembly (hg18)

^b^SOX10 consensus sequences are indicated in bold, underlined text

### The SOX10 consensus sequence is required for the orientation-independent activity of three regulatory elements at *SOX5*, *SOX6*, and *NFIB*

**Fig. 2:**
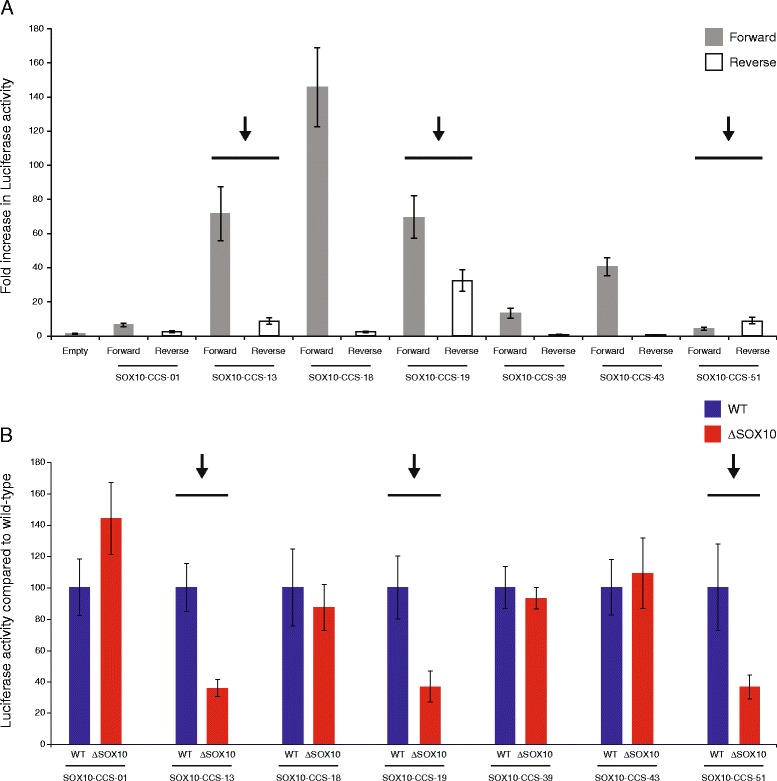
Three genomic segments require the SOX10 consensus sequence for activity in Schwann cells. **a** The seven active regions from Fig. 1 were tested in forward and reverse orientation in rat Schwann (S16) cells. Luciferase data are expressed relative to a control vector without a genomic segment (‘Empty’). Error bars indicate standard deviations and arrows and lines indicate genomic segments that are active in both orientations. **b** Luciferase reporter gene constructs containing either the wild-type sequence (WT) or the sequence lacking the SOX10 consensus sequence(s) (ΔSOX10) were transfected into S16 cells and tested in luciferase assays. The luciferase activity associated with each ΔSOX10 construct is expressed relative to the respective wild-type construct. Error bars indicate standard deviations and arrows and lines indicate genomic segments with a required SOX10 consensus sequence

To determine if the regulatory activity of the seven genomic segments is dependent on the orientation of the DNA sequence, we retested the activity of each segment in both the ‘forward’ and ‘reverse’ orientation relative to a construct with no genomic insert (‘Empty’) within our reporter gene construct in S16 cells. This revealed three genomic segments that enact a greater than 2.5-fold increase in luciferase activity in both orientations (Fig. 2a): SOX10-CCS-13 (72-fold forward and 9-fold reverse), SOX10-CCS-19 (70-fold forward and 33-fold reverse), and SOX10-CCS-51 (4-fold forward and 9-fold reverse). To assess the specificity of these results to Schwann cells, we tested each of the seven genomic segments in both orientations in cultured mouse motor neurons (MN1 cells) [23], which do not express endogenous SOX10 [19]. None of the genomic segments enact a greater than 2.5-fold increase in luciferase activity in both orientations in MN1 cells suggesting that our data in S16 cells is Schwann-cell specific; however, three had low levels of activity in only the forward orientation in MN1 cells (Additional file 6: Figure S1): SOX10-CCS-39 (5.5-fold), SOX10-CCS-43 (6.7-fold), and SOX10-CCS-51 (4-fold).

To test the necessity of the conserved SOX10 consensus sequence for the observed activity associated with the seven genomic segments described above, we deleted the dimeric SOX10 site along with the intervening sequence in each construct (ΔSOX10) and compared the activity to the wild-type genomic segment using the more active orientation. This revealed three genomic segments that display at least a 50 % reduction in activity upon deleting the SOX10 consensus sequence (Fig. 2b): SOX10-CCS-13, SOX10-CCS-19, and SOX10-CCS-51. Combined, our data are consistent with these three genomic segments—at the *SOX6*, *SOX5*, and *NFIB* loci, respectively—representing Schwann cell enhancers that harbor functional SOX10 binding sites.

### SOX10 is required for the activity of the three regulatory elements at *SOX5*, *SOX6*, and *NFIB*

**Fig. 3:**
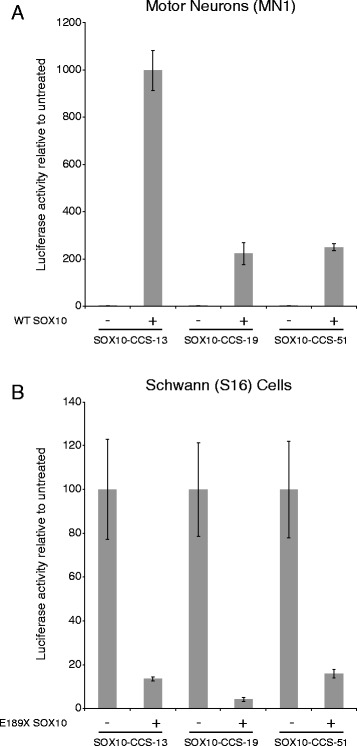
SOX10 is required for the regulatory activities of SOX10-CCS-13, SOX10-CCS-19 and SOX10-CCS-51. **a** Luciferase reporter gene constructs harboring SOX10-CCS-13, SOX10-CCS-19 or SOX10-CCS-51 were transfected into mouse motor neurons (MN1) with or without a construct to express wild-type SOX10. The luciferase activity associated with each construct in the presence of SOX10 is expressed relative to that of the construct in the absence of SOX10. **b** Luciferase reporter gene constructs harboring SOX10-CCS-13, SOX10-CCS-19 or SOX10-CCS-51 were transfected into rat Schwann (S16) cells with or without a construct to express dominant-negative (E189X) SOX10. The luciferase activity associated with each construct in the presence of E189X SOX10 is expressed relative to that of the construct in the absence of E189X SOX10. Error bars indicate standard deviations in both panels

To test if SOX10 induces the activity of SOX10-CCS-13, SOX10-CCS-19, and SOX10-CCS-51, we co-transfected each reporter gene construct with or without a construct to express wild-type SOX10 in MN1 cells, which do not express endogenous SOX10 [8, 19]. Subsequently, we compared the activity of each construct in the presence or absence of SOX10 expression. There was a ~1,000-fold increase in the activity of SOX10-CCS-13 and a ~200-fold increase in the activities of SOX10-CCS-19 and SOX10-CCS-51 in the presence of SOX10 (Fig. 3a).

SOX8, SOX9, and SOX10 belong to the SOXE family of transcription factors, which bind to nearly identical sequence motifs [24]. To test if SOX10 specifically regulates SOX10-CCS-13, SOX10-CCS-19, and SOX10-CCS-51, we co-transfected each reporter construct with or without a construct to express SOX8 or SOX9 in MN1 cells [19, 25] and compared the effect on regulatory activity with that induced by SOX10. In the presence of SOX8 we observed a ~140-fold, ~75-fold, and ~50 fold increase in the activity of SOX10-CCS-13, SOX10-CCS-19, and SOX10-CCS-51, respectively (Additional file 7: Figure S2). In the presence of SOX9 we observed a ~350-fold, ~150-fold, and ~80-fold increase in the activity of SOX10-CCS-13, SOX10-CCS-19, and SOX10-CCS-51, respectively (Additional file 7: Figure S2). Importantly, SOX8 and SOX9 did not increase the luciferase activity of these regions to the same level as SOX10 (Additional file 8: Figure S3), suggesting that SOX10 has a higher affinity for the sequences within SOX10-CCS-13, SOX10-CCS-19, and SOX10-CCS-51.

SOX10 is known to synergistically interact with other transcription factors to enact gene expression [OCT6, BRN2, and EGR2 in Schwann cells, OLIG2 and MYRF in oligodendrocytes [26], and PAX3 and MITF in melanocytes [27]]. Thus, we wanted to determine if the most well-characterized co-factor in Schwann cells (EGR2) works synergistically to activate these elements. EGR2, a master regulator of Schwann cell myelination, is regulated by SOX10, OCT6, and BRN2 [28]. SOX10 and EGR2 synergistically regulate key myelin genes such as *PMP22* [10], *MPZ* [11], and *GJB1* [9]. We co-transfected SOX10-CCS-13, SOX10-CCS-19, and SOX10-CCS-51 reporter constructs with a construct to express EGR2 and SOX10 in MN1 cells and compared the effect on regulatory activity with that induced by SOX10 alone (Additional file 8: Figure S3). In the presence of EGR2 we observed a moderate increase in luciferase activity of SOX10-CCS-13 (~2.2-fold), SOX10-CCS-19 (~12-fold) and SOX10-CCS-51 (~10-fold) (Additional file 8: Figure S3). However, in the presence of both EGR2 and SOX10 we did not see an increase in activity above that induced by SOX10 alone (even though an equivalent amount of SOX10 expression vector was transfected in each experiment). These data suggest that the three regions are primarily regulated by SOX10 and that EGR2 and SOX10 do not act synergistically upon them.

To determine if SOX10 is necessary for the activity of SOX10-CCS-13, SOX10-CCS-19, and SOX10-CCS-51 in Schwann cells, S16 cells were transfected with each SOX10-CCS luciferase reporter gene construct along with a construct to express a dominant-negative mutant form of SOX10 (E189X), which interferes with the function of endogenous SOX10 [8]. Importantly, E189X SOX10 has been shown to specifically reduce the activity of genomic segments harboring SOX10 binding sites in luciferase assays [29]. We observed a greater than 85 % reduction in the activity of all three genomic segments upon co-transfection with E189X SOX10 (Fig. 3b). Combined, our data indicate that SOX10 is required for the in vitro enhancer activity of SOX10-CCS-13, SOX10-CCS-19, and SOX10-CCS-51.

### SOX10-CCS-13 is a previously unreported, alternative *Sox6* promoter

**Fig. 4:**
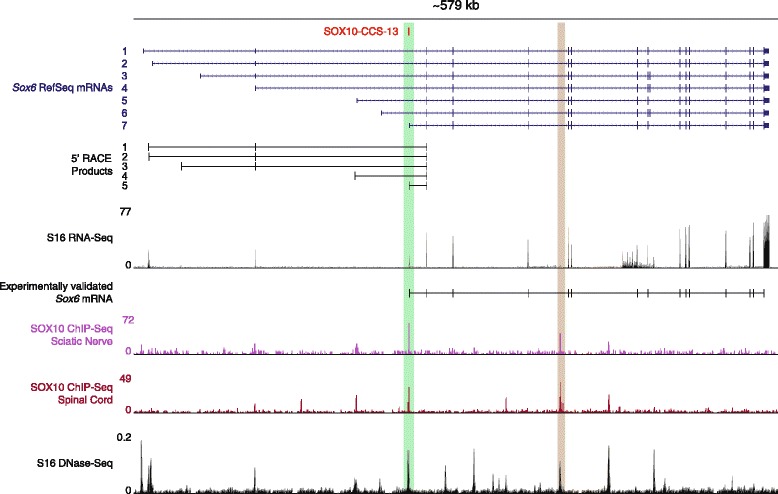
SOX10-CCS-13 is an alternative promoter at the *Sox6* locus. The ~579 kb rat *Sox6* locus is shown on the UCSC Rat Genome Browser. SOX10-CCS-13 is indicated in red along with the seven human, mouse, and rat *SOX6* RefSeq mRNAs (blue). *Sox6*-specific 5’ RACE was performed on RNA from S16 cells and the five distinct *Sox6* sequences were mapped to the rat genome. Please note that SOX10-CCS-13 maps to both the 5’ end of the seventh *Sox6* mRNA and the fifth unique 5’ RACE-generated sequence. RNA-Seq data from S16 cells were mapped to *Sox6* (the y-axis indicates sequence read depth) as was a PCR-amplified, full-length mRNA that contains *Sox6* exon 1G. Genome-wide regulatory marks were also mapped to *Sox6* with the Y-axes indicating normalized sequence read depths (both SOX10 ChIP-seq data sets) and F-Seq scores (DNase-seq). The green highlighted region marks the general position of SOX10-CCS-13 across all data sets and the brown highlighted region marks another potential SOX10 response element at *Sox6*

Examination of SOX10-CCS-13 on the UCSC Genome Browser revealed that the SOX10 consensus sequence within this genomic segment is also conserved between human and zebrafish (data not shown) further suggesting an important role for this SOX10 response element in jawed vertebrates. Therefore, to validate our computational approach we pursued additional analyses of SOX10-CCS-13, which resides at the *SOX6* locus. Closer scrutiny of the *SOX6* locus on the UCSC Genome Browser [30] revealed seven unique *SOX6* mRNA isoforms in human, mouse, or rat, distinguished by alternative, non-coding first exons. Interestingly, SOX10-CCS-13 maps directly upstream of the 3’-most alternative first exon, which we named *SOX6* exon 1G (Fig. 4). We therefore hypothesized that SOX10-CCS-13 acts as an alternative promoter at *SOX6*. To test this, we performed 5’-rapid amplification of cDNA ends (5’-RACE). Briefly, a cDNA library was generated using RNA isolated from cultured rat Schwann (S16) cells and a reverse primer in exon 5 of the rat *Sox6* gene. Subsequently, nested PCR was performed using reverse primers in exon 4 and then exon 3 of *Sox6*. The PCR products were cloned, sequenced, and aligned to the rat *Sox6* locus. These analyses revealed the presence of five unique *Sox6* transcription start sites in cultured Schwann cells with 14 of the 44 *Sox6*-specific sequences mapping directly downstream of SOX10-CCS-13 (Fig. 4). Analysis of RNA-seq data generated in S16 cells (Antonellis and Law, in preparation) also revealed reads that map to *Sox6* exon 1G (Fig. 4), with split reads into downstream exons, but no split reads into upstream exons (data not shown). Additionally, we were able to amplify and sequence-verify a full length *Sox6* mRNA that originates at exon 1G in S16 cells (Fig. 4).

To assess the in vivo relevance of SOX10-CCS-13 we analyzed recently published SOX10 ChIP-seq data performed on nuclei isolated from rat spinal cord and sciatic nerve [14, 15]. Furthermore, to establish that this genomic segment resides in open chromatin we performed DNase-seq analysis on nuclei isolated from cultured rat Schwann (S16) cells (see below). The SOX10 ChIP-seq analyses revealed that SOX10 binds to SOX10-CCS-13 in relevant tissues in vivo and the DNase-seq experiment revealed that this genomic segment resides in open chromatin in cultured Schwann cells (Fig. 4, green box). Combined, these data support our conclusion that SOX10-CCS-13 is a SOX10 response element in Schwann cells. Interestingly, these genome-wide functional studies revealed additional SOX10 binding sites at *Sox6* in vivo further supporting the notion that *Sox6* is a SOX10 target gene (*e.g.*, brown highlighted region in Fig. 4). Combined, our data indicate that SOX10-CCS-13 represents an internal, alternative, SOX10-responsive promoter at *Sox6*.

### SOX10 is necessary and sufficient for the expression of *Sox6* transcripts harboring exon 1G

**Fig. 5:**
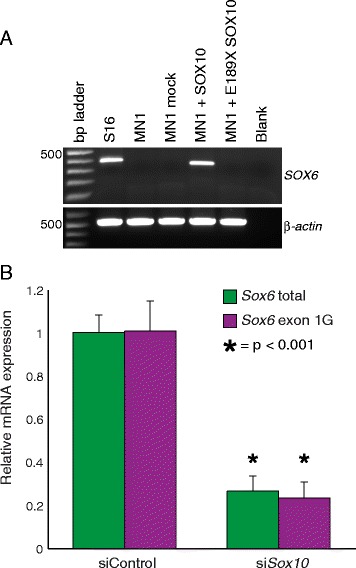
SOX10 is necessary and sufficient for *SOX6* expression. **a** RT-PCR was performed to detect the expression of *Sox6* transcripts harboring exon 1G using cDNA isolated from S16 cells, MN1 cells, or MN1 cells transfected with no expression construct (mock) or a construct to express wild-type or dominant-negative (E189X) SOX10. Base pair (bp) ladders are indicated on the left. RT-PCR for *β-actin* and samples including no cDNA (‘Blank’) were employed as positive and negative controls, respectively. Please note that while the same primers were used for each reaction, the rat (S16) PCR product was 402 base pairs and the mouse (MN1) PCR product was 349 bp; the rat genome harbors a 53 base pair rat-specific insertion, which we confirmed via DNA sequence analysis. **b** Rat Schwann (S16) cells were treated with a control siRNA (left side) or a siRNA targeted against *Sox10* (right side). Quantitative RT-PCR was used to measure expression levels of total *Sox6* (green bars) or *Sox6* exon 1G-containing (purple bars) transcripts. Error bars indicate standard deviations

To determine if SOX10 is sufficient to direct the expression of *Sox6* transcripts, we performed RT-PCR using primers designed in *Sox6* exon 1G and exon 2 in regions conserved between rat and mouse. While these primers amplify *Sox6* transcripts containing exon 1G from a cDNA library generated from S16 RNA, we were not able to amplify these transcripts from a cDNA library generated from cultured mouse motor neurons (MN1 cells), which do not express endogenous SOX10 (Fig. 5a). However, when MN1 cells were transfected with a construct to express wild-type SOX10, *Sox6* transcripts containing exon 1G were detected and verified by DNA sequence analysis. Mock transfection or transfection with a construct to express a non-functional mutant version of SOX10 (E189X) [8] did not allow amplification of *Sox6* transcripts containing exon 1G (Fig. 5a). Thus, SOX10 is sufficient to activate the expression of *Sox6* transcripts harboring exon 1G in MN1 cells.

To determine if SOX10 is necessary for the expression of *Sox6* transcripts containing exon 1G in Schwann cells, we treated S16 cells with a previously validated siRNA against *Sox10* [15, 20] and tested for an effect on total *Sox6* mRNA levels and for an effect on the level of transcripts containing exon 1G. This analysis revealed a ~70 % decrease in both total *Sox6* expression and in the expression of transcripts containing exon 1G (Fig. 5b), consistent with SOX10 regulating the promoter activity of SOX10-CCS-13 in Schwann cells. Combined, our data indicate that SOX10 is both necessary and sufficient for the expression of *Sox6* mRNA isoform 7 (Fig. 4) in our in vitro cell culture model systems.

### SOX10 regulates the expression of genes that inhibit myelination

Our stringent computational and functional analyses rapidly identified a previously unreported SOX10-responsive promoter at the *SOX6* locus. Importantly, this finding was facilitated by the knowledge of a well-defined SOX10 consensus sequence and reports that SOX10 binding sites can be conserved among vertebrate species including human and chicken [18–20]. To determine if our conservation analysis combined with whole genome datasets can reveal a set of high-confidence SOX10 response elements for further study we: (1) utilized available SOX10 ChIP-seq data generated from rat Schwann cell nuclei in vivo [14]; (2) performed DNase-seq on cultured rat Schwann (S16) cell nuclei; and (3) identified 67,482 non-coding SOX10 monomeric consensus sequences conserved between human, mouse, and chicken (data not shown), and converted them to the rat genome [rn5; 61,133 (90.5 %) were successfully converted]. Intersecting these three data sets revealed 214 rat genomic segments that harbor conserved SOX10 consensus sequences and that map to SOX10 ChIP-seq and DNase-seq peaks (Additional file 9; these genomic segments were computationally extracted as SOX10 ChIP-seq peaks). To determine if this approach identified specific biological pathways, we extracted the name of the rat RefSeq gene closest to each region—the 214 genomic segments map to 191 known genes (Additional file 9)—and performed a gene ontology search using the overrepresentation test for biological processes (geneontology.org). This analysis revealed 183 biological processes with a *p*-value less than 0.05 and 37 biological processes that showed a greater than five-fold enrichment compared to the human genome. Ten of the identified biological processes directly relate to myelinating glia, which all resided in the top 14 enriched terms (Table 2). Therefore, this combined strategy provided a highly confident set of 214 SOX10-response elements at 191 loci for future functional studies aimed at better understanding the biological process of myelination (Additional file 9).

**Table 2:** Gene ontology annotations of loci harboring conserved SOX10 binding sites

GO biological process complete	Human	Our list	Expected	*P*-value	Loci
Negative regulation of oligodendrocyte differentiation	12	4	0.1	2.74E-02	*HES1, ID2, NOTCH1, ID4*
Negative regulation of glial cell differentiation	25	6	0.21	6.34E-04	*HES1, ID2, NOTCH1, HMGA2, MYCN, ID4*
Regulation of astrocyte differentiation	25	6	0.21	6.34E-04	*HES1, ID2, NOTCH1, HMGA2, MYCN, ID4*
Regulation of oligodendrocyte differentiation	28	5	0.23	3.29E-02	*HES1, TCF7L2, ID2, NOTCH1, ID4*
Negative regulation of gliogenesis	34	6	0.28	3.77E-03	*HES1, ID2, NOTCH1, HMGA2, MYCN, ID4*
Oligodendrocyte differentiation	60	10	0.5	9.43E-07	*SOX6, NTRK2, PTPRZ1, SOX8, SOX10, TCF7L2, ID2, NOTCH1, SOX5, ID4*
Regulation of gliogenesis	74	10	0.61	6.96E-06	*PTPRZ1, SOX8, HES1, SOX10, TCF7L2, ID2, NOTCH1, HMGA2, MYCN, ID4*
Regulation of glial cell differentiation	54	7	0.45	3.23E-03	*HES1, TCF7L2, ID2, NOTCH1, HMGA2, MYCN, ID4*
Glial cell differentiation	135	13	1.12	1.22E-06	*SOX6, PTPRZ1, NTRK2, SOX8, HES1, SOX10, TCF7L2, ID2, NOTCH1, PPAP2B, SOX5, ID4, PARD3*
Gliogenesis	168	13	1.39	1.66E-05	*SOX6, PTPRZ1, NTRK2, SOX8, HES1, SOX10, TCF7L2, ID2, NOTCH1, PPAP2B, SOX5, ID4, PARD3*

**Fig. 6:**
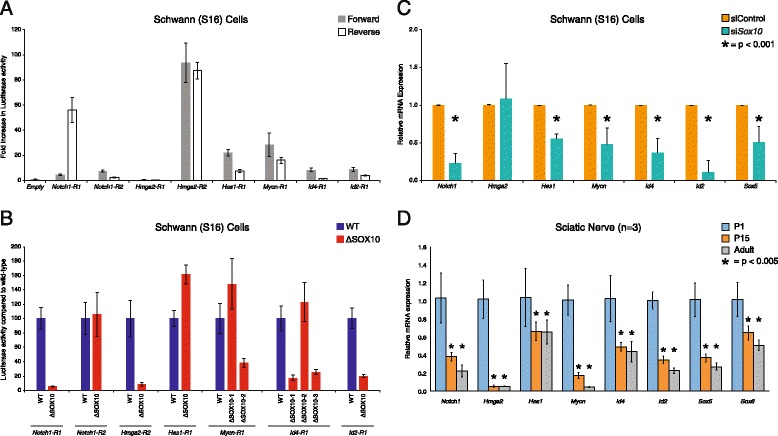
SOX10 regulates the expression of genes that inhibit glial cell differentiation. **a** Eight genomic segments at the rat *Notch1*, *Hmga2*, *Hes1*, *Mycn*, *Id4*, and *Id2* loci were cloned upstream of a luciferase reporter gene in both the forward and reverse orientations and tested for luciferase activity in rat Schwann (S16) cells. Luciferase data are expressed relative to a control vector with no genomic insert (‘Empty’). Error bars represent standard deviations. **b** The conserved SOX10 consensus sequence(s) were deleted in each of the seven regions that were active in **a** (see text for details). Luciferase reporter gene constructs containing the wild-type sequence (WT) or the sequence lacking the SOX10 consensus sequence(s) (ΔSOX10) were transfected into S16 cells and luciferase assays performed. Luciferase activities are expressed relative to the wild-type expression constructs and error bars represent standard deviations. **c** Rat Schwann (S16) cells were treated with a control siRNA or a siRNA targeted against *Sox10*. Quantitative RT-PCR was used to measure expression levels of each indicated gene. Asterisks indicate a p-value smaller than 0.001 and error bars indicate standard deviations. **d** RNA was purified from three independent rat sciatic nerves at the P1, P15, and adult timepoints. Quantitative RT-PCR was used to measure expression levels of each indicated gene with values expressed relative to expression levels at P1. Asterisks indicate a p-value smaller than 0.005 and error bars indicate standard deviations

Interestingly, three of the 10 gene ontology biological processes that relate to myelination specifically relate to negative regulation of gliogenesis, which was due to the presence of six genes: *NOTCH1, HMGA2*, *HES1*, *MYCN*, *ID4*, and *ID2* (Table 2). Computational analyses revealed eight SOX10 consensus sequences within DNase-seq and SOX10 ChIP-seq peaks at these six loci (Table 3). To determine if *NOTCH1*, *HMGA2*, *HES1*, *MYCN*, *ID4*, and *ID2* harbor *bona fide* SOX10 response elements, we amplified genomic regions surrounding the SOX10 consensus sequences using rat genomic DNA and cloned each genomic segment (in both the ‘forward’ and ‘reverse’ orientation) upstream of a minimal promoter directing luciferase expression. The regulatory activity of each genomic segment was tested in S16 cells as described above. This revealed five genomic segments (‘regions’ or ‘R’) that directed reporter gene activity at least 2.5-fold higher than the empty control vector in both orientations: *Notch1-R1* (4.7-fold forward and 56-fold reverse), *Hmga2-R2* (93.7-fold forward and 87-fold reverse), *Hes1-R1* (22-fold forward and 7.6-fold reverse), *Mycn-R1* (28-fold forward and 16-fold reverse) and *Id2-R1* (8.9-fold forward and 4.1-fold reverse) (Fig. 6a). Regions *Notch1-R2* (7.6-fold) and *Id4-R1* (8.6-fold) directed reporter gene activity at least 2.5-fold higher than the empty control vector only in the forward orientation (Fig. 6a). *Hmga2-R1* was not active in either orientation and was excluded from further analysis. Thus, we identified seven genomic sequences at six loci (*NOTCH1*, *HMGA2*, *HES1*, *MYCN*, *ID4*, and *ID2*) that display regulatory activity in Schwann cells.

**Table 3:** Eight genomic segments within loci that inhibit glial cell differentiation

Element ID	UCSC Coordinates^a^	SOX10 Consensus Sequence^b^
*Notch1-R1*	chr3:9,307,836-9,308,296	**ACAAT**GGGGCC**TCTGT**
*Notch1-R2*	chr3:9,308,175-9,309,096	**ACAAT**CGGC**TTTGT**
*Hmga2-R1*	chr7:65,390,088-65,391,287	CTTAG**ACACA**GCACTT
*Hmga2-R2*	chr7:65,427,912-65,428,606	**ACACA**GGCCCCTC**TTTGT**
*Hes1-R1*	chr11:77,415,315-77,415,779	**TGTGT**GAGCGCCA**TGTGT**
*Mycn-R1*	chr6:51,229,947-51,230,533	**ACAAT**GGCCTC**TTTCT**ACAG**ACAAT**
*Id4-R1*	chr17:18,701,460-18,702,118	**ACAAA**AACAGCAGTAAATGGAGGCC**TTTGT**
*Id2-R1*	chr6:53,090,794-53,091,254	**ACAAG**AAACAC**ATTGT**

^a^Coordinates refer to the March 2012 UCSC Genome Browser Rat assembly (rn5)

^b^SOX10 consensus sequences are indicated in bold, underlined text

To determine if the identified SOX10 consensus sequences (Table 3) are important for the regulatory activity of the seven active regions described above (Fig. 6a) we deleted the SOX10 consensus sequence from each construct (termed ‘ΔSOX10’ in Fig. 6b) and compared the activity to the wild-type construct using the more active orientation. *Notch1-R1*, *Notch1-R2*, *Hmga2-R2*, *Hes1-R1*, and *Id2-R1* contain dimeric SOX10 consensus sequences, which were deleted along with the intervening sequence. *Mycn-R1* contains a monomeric consensus sequence (ΔSOX10-1) and a dimeric consensus sequence (ΔSOX10-2), which were independently deleted. *Id4-R1* contains a dimeric consensus sequence with a 20 base-pair intervening sequence. Since this intervening sequence is longer than those previously observed for validated dimeric SOX10 binding sites [16, 18–20, 29, 31] we studied each monomer independently. Specifically, we deleted the dimeric consensus sequence along with intervening sequence (ΔSOX10-1), the first monomer only (ΔSOX10-2), and the second monomer only (ΔSOX10-3). Deleting the SOX10 consensus sequences in regions *Notch1-R1*, *Hmga2-R2*, *Mycn-R1* (ΔSOX10-2), *Id4-R1* (ΔSOX10-1 and ΔSOX10-3), and *Id2-R1* reduced luciferase activity in S16 cells by at least 50 % (Fig. 6b), indicating that the SOX10 consensus sequences in these five regions are important for their regulatory activity. In contrast, deleting the SOX10 consensus sequences in *Notch1*-*R2* and *Hes1*-*R1* did not reduce the enhancer activity associated with these genomic segments (Fig. 6b).

We have shown that SOX10 directly regulates *SOX6* in Schwann cells (see above). To determine if SOX10 positively regulates the expression of *Notch1*, *Hmga2*, *Hes1*, *Mycn*, *Id4*, *Id2*, and *Sox5* in cultured rat Schwann (S16) cells we again utilized the *Sox10* siRNA that has been shown to efficiently down-regulate *Sox10* expression [15, 20]. After isolation of mRNA at 24 h post-transfection, qRT-PCR shows that *Sox10* depletion in S16 cells results in the reduced expression of all of the above genes except for *Hmga2* (Fig. 6c). Similar findings—including the absence of reduced *Hmga2* expression—were observed upon repressing SOX10 function in vivo using primary Schwann cells; however, this system is prone to variability due to heterogeneous cell populations (Additional file 10: Figure S4).

To directly test if *NOTCH1*, *HMGA2*, *HES1*, *MYCN*, *ID4*, *ID2*, *SOX5*, and *SOX6* are developmentally regulated during myelination *in vivo* we examined mRNA levels at three timepoints in rat sciatic nerve (*n* = 3 at each timepoint). P1 corresponds to the onset of myelination, P15 is a peak timepoint of myelination in the PNS, and adult sciatic nerve is a timepoint where active myelination has subsided. Interestingly, the expression of all seven genes tested (*Notch1*, *Hmga2*, *Hes1*, *Mycn*, *Id4*, *Id2*, *Sox5*, and *Sox6*) are highest at P1 and then repressed at P15 and adult, consistent with a role in repressing precocious myelination (Fig. 6d).

## Discussion

Previous efforts have identified SOX10 binding sites and target genes in Schwann cells [14, 15]. These efforts, and others [32], have utilized a variety of experimental methodologies to identify putative SOX10 regulatory elements across diverse SOX10-positive tissues. While each approach uncovered novel SOX10 response elements, no single method has been successful in the identification of all SOX10 response elements. While the strategies presented here also are unable to fully capture all SOX10 binding sites, the combination of multiple datasets and methodologies generally yields a stronger predictive power for identifying regulatory regions compared to any one individual method [33]. Indeed, when we combined our DNase-seq data with previously generated SOX10 ChIP-seq data [14], we were able to quickly prioritize and validate novel SOX10 response elements near genes with a known role in myelination. While previous efforts to identify SOX10 response elements focused on the required function of SOX10 in cultured cells or tissues at specific developmental stages, our computational approach utilizes sequence conservation to identify putative SOX10 regulatory regions throughout the genome in a tissue-independent manner. The combination of our less-biased (albeit less biologically relevant) computational approach with DNase-seq and ChIP-seq is likely the reason that we were able to identify specific repressors of myelination as putative SOX10 target genes (*e.g.*, these would not have been identified in myelinating Schwann cells). As such, we feel that the datasets generated here will be useful to investigators studying comparative genomics, SOX protein function, and Schwann cell biology. First, the conserved sequences we identified could be used to similarly prioritize consensus sequences for other transcription factors important for vertebrate development. Second, the SOX10 consensus sequences we identified could be used to prioritize putative binding sites in other SOX10-positive cells including oligodendrocytes, melanocytes, and developing enteric nervous system neurons [34]. Finally, our DNase-seq data from rat Schwann (S16) cells will be useful for anyone studying transcriptional regulatory elements, highly expressed genes, or any other nuclear structure characterized by open chromatin in myelinating Schwann cells; S16 cells express many myelin-related genes (*e.g.*, *PMP22*, *MPZ*, *MBP*, and *MAG*) and transcription factors (*e.g.*, SOX10 and EGR2) and are biochemically similar to myelinating Schwann cells [35].

The analyses performed here may also provide insight into the nucleotide requirements for a SOX10 response element. We functionally evaluated a total of 62 conserved, genomic segments that harbor a predicted dimeric SOX10 binding site. Interestingly, simply cloning a highly conserved dimeric SOX10 consensus sequence upstream of a minimal promoter was not enough to enact regulatory activity in cultured Schwann cells as evidenced by the 50 genomic segments with no (or very low) regulatory activity in Fig. 1. While there are many possible explanations for this finding, one is that there are nucleotide-specific requirements for the intervening sequences (*i.e.*, the nucleotides between the head-to-head monomeric sites). To assess this, we compared the length and GC content of all 62 dimeric sites to those dimeric sites that were both active and required for the observed regulatory activity (*n* = 7). While there was no significant difference in the average intervening sequence length between the two groups, the seven active sites all had intervening sequence lengths between five and eight nucleotides consistent with previous reports that six basepairs provides the ideal spacing between monomers [16]. Interestingly, there was a marked difference in the GC content when comparing the total population of intervening sequences (GC content = 35 %) to the intervening sequences in the active dimeric sites (GC content = 61 %). These data are consistent with the high GC content of the intervening sequences within previously validated dimeric SOX10 binding sites [13, 18–20, 29] and with a ‘G’ nucleotide being the most commonly observed nucleotide after the core motif [14]. Thus, future predictions of dimeric SOX10 binding sites should allow for high GC content and five to eight basepairs between the head-to-head consensus sequences.

Our efforts predicted eight putative SOX10 target loci with a known role in repressing glial cell differentiation: *NOTCH1*, *HMGA2*, *HES1*, *MYCN*, *ID2*, *ID4, SOX5, and SOX6*. These findings were unexpected due to the known role of SOX10 in regulating the expression of genes that encode myelin proteins (*e.g.*, *MBP*, *MPZ*, and *PMP22*) [10, 12, 13, 31, 36, 37]. We showed that all eight loci are developmentally regulated during myelination *in vivo* in a manner consistent with a role in inhibiting glial cell differentiation. We were also able to functionally validate a SOX10 binding site at seven of the eight loci. We identified a SOX10 ChIP-seq peak at *HES1* and luciferase assays demonstrated that this genomic segment has strong enhancer activity (Fig. 6a). However, deletion of the predicted SOX10 binding sites in *Hes1-R1* (Table 3) did not reduce luciferase activity. Further mutagenesis of this genomic segment will be required to identify sequences necessary for the observed activity, which may reveal a degenerate SOX10 consensus sequence. When we depleted SOX10 activity in Schwann cells in vitro and in vivo seven of the eight loci were down-regulated; while *HMGA2* harbors a validated SOX10 response element (Fig. 6b), depletion of SOX10 activity did not reduce *Hmga2* expression. Further analysis will be required to determine if the SOX10 response element at *Hmga2* regulates an adjacent locus or if depletion of SOX10 at specific developmental timepoints results in reduced *Hmga2* expression. Consistent with our findings, previous global analyses of SOX10 function revealed that two of the above eight loci are downstream of SOX10: *Id2* and *Notch1* [14]; our analysis now localizes at least some of the SOX10-dependent enhancers responsible for the regulation of these two loci.

SOX5 and SOX6 are members of the SOXD family of transcription factors and act as negative regulators of myelination in the central nervous system [38]; these proteins, which do not have transactivation or transrepression domains [39], inhibit the expression of SOX10 target genes (*e.g.*, *MBP*) in oligodendrocytes by competing with SOX10 for DNA binding at sites within cis-acting regulatory elements. To allow oligodendrocyte differentiation and myelin production, *SOX6* mRNA is targeted for degradation by two microRNAs (miR) in these cells: miR-219 and miR-338 [40]. It was recently reported that SOX13 (the third and final member of the SOXD subgroup) also has an antagonistic effect on the ability of SOX10 to activate the expression of myelin genes in the central nervous system [41]. Indeed, *SOX13* is among the group of 191 loci at which we identified a highly confident SOX10 binding site: a single, conserved genomic segment within SOX10 ChIP-seq and DNase-seq peaks ~62 kb upstream of *Sox13* (rn5 coordinates chr13:55425486–55425636) was identified (Additional file 9). Interestingly, a relationship between SOXD and SOXE (SOX8, SOX9, and SOX10) transcription factors has been proposed since ablation of SOX8 or SOX9 (but not SOX10) reduces *Sox6*, but not *Sox5*, expression in the developing spinal cord [38].

In addition to genes encoding SOXD proteins, our studies predict that *NOTCH1*, *HES1*, *MYCN*, *ID2*, and *ID4* are SOX10 target genes. NOTCH1 is a transmembrane receptor that regulates Schwann cell proliferation and inhibits Schwann cell differentiation in perinatal nerves, and facilitates dedifferentiation of Schwann cells after nerve injury [42]. HES1 is an effector of NOTCH signaling, acts as a transcriptional repressor [43, 44], and is highly expressed during early stages of Schwann cell development [42]. In cultured mouse oligodendrocytes, HES1 maintains cells in an immature state and overexpression of HES1 results in reduced expression of myelin related genes (*Mbp* and *Plp*) [45]. MYCN is a proto-oncogene and is known to inhibit astrocyte differentiation from neural precursor cells [46]; however, the role of MYCN during Schwann cell myelination has not been studied. Inhibitors of differentiation 2 and 4 (ID2 and ID4) are known to inhibit oligodendrocyte differentiation and the lack of both proteins results in premature oligodendrocyte differentiation [47–49]. Furthermore, *Id2* and *Id4* expression declines in Schwann cell development and ID2 limits induction of *myelin protein zero* expression in primary Schwann cells [50, 51]. Consistent with our findings, RNA-seq of oligodendrocytes isolated at various stages of mouse brain development [52] show that *Sox5*, *Sox6*, *Notch1*, *Hes1*, *Mycn*, *Id2*, and *Id4* are developmentally regulated in the central nervous system—*Hmga2* does not appear to be expressed in the cells assessed in that study. Therefore, these genes likely play a role in preventing premature glial cell differentiation in both the central and peripheral nervous systems.

**Fig. 7:**
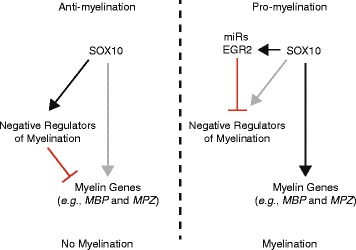
A model for the role of SOX10 in maintaining a pre-myelinating state. Previous to myelination (anti-myelination; left side), SOX10 activates the expression of negative regulators of myelination, which inhibit the expression of myelin genes such as *MBP* and *MPZ*. During activation of the myelination program (pro-myelination; right side), EGR2 and micro RNAs (miRs) inhibit the expression of negative regulators of myelination, which allows SOX10 (and EGR2) to positively regulate the expression of myelin genes

Combined with previous findings, our data predict a model (Fig. 7) where SOX10 activates the expression of genes that inhibit Schwann cell differentiation, possibly during early stages of Schwann cell development, thus preventing the precocious expression of myelin proteins. Subsequently, EGR2, NAB, and microRNAs are known to inhibit the expression of the negative regulators of myelination (*e.g.*, SOXD proteins), which would allow the expression of myelin proteins. In addition to the data presented in this study, previous reports support specific aspects of this model. For example, EGR2 likely represses the expression of many of the eight loci reported here. EGR2 and NAB repress *ID2* and *ID4* before myelination via NAB binding to CHD4 [51] [conditional ablation of CHD4 in Schwann cells causes increased expression of immature Schwann cell genes including *ID2* and delayed myelination, radial sorting defects, hypomyelination, and the persistence of promyelinating Schwann cells in conditional knockout mice [53]]. Furthermore, a comparison of SOX10 and EGR2 binding with expression profiles in Schwann cells treated with siRNA for SOX10 and EGR2-deficent peripheral nerves [14] revealed that *NOTCH1* and *ID2* are SOX10-activated and EGR2-repressed, and *ID2*, *HMGA2*, *SOX5*, and *ID4* remain high in peripheral nerves from *Egr2- or Nab-deficient* mice [51, 54]. Finally, SOX10 directly regulates the expression of *EGR2* [28] and miR-338 [20]. In sum, our findings suggest that SOX10 has a role in maintaining a premyelinating state during non-myelinating stages of Schwann cell development.

## Conclusions

In this study, we developed a computational and functional pipeline to expand the panel of known SOX10 response elements and target loci. These efforts predict a novel function for SOX10 in repressing myelination in early stages of Schwann cell (and possibly neural crest) development. Furthermore, we provide useful datasets for the scientific community and expand the mutational screening space for disease-causing mutations—or modifiers of disease—in patients with peripheral neuropathy and other SOX10-related phenotypes.

## Methods

### Computational identification and prioritization of SOX10 consensus sequences

To identify all SOX10 consensus sequences in the human genome we downloaded individual text files for each human chromosome (hg18) from the UCSC Human Genome Browser [30] and wrote a Perl script (available upon request) that examines each file for the consensus sequences (using a regular expression analysis) ‘ACACA’ or ‘ACAAD’; where ‘D’ is a G, T, or A nucleotide. To identify two SOX10 consensus sequence monomers that are oriented in a head-to-head manner (and that may represent a dimeric SOX10 binding site) we wrote a second Perl script that examines the human chromosome text files and reports each ‘ACACA’ or ‘ACAAD’ consensus sequence that is five to 10 base pairs 5’ to the reverse complement of this consensus sequence: ‘TGTGT’ or ‘HTTGT’, where ‘H’ represents an A, C, or T nucleotide.

To identify genomic sequences that are identical between human, mouse, and chicken we downloaded the vertebrate (44 species) multiz alignment (maf) files from the UCSC Human Genome Browser (hg18) and extracted the alignments for human, mouse, and chicken. Next, we utilized the program ExactPlus [55] to identify all human sequences that are at least 5 base pairs long and identical between all three species. All subsequent computational analyses that assess for overlap between these and other datasets were performed using the UCSC Table Browser [56] and the ‘intersection’ tool. For these analyses we employed UCSC Genome custom tracks containing each: (1) human RefSeq (hg18) protein-coding sequence to exclude coding sequences; (2) human RefSeq entry (hg18) plus 2.5 kb upstream and 2.5 kb downstream of the transcriptional unit to identify regions that map to known genes; (3) SOX10 ChIP-seq peak in the rat genome (rn5) using HOMER analysis [57] (Additional file 11) of previously described P15 sciatic nerve data sets; and (4) DNase-seq peak in the rat genome (rn5) that has an F-Seq [58] score of at least 0.08 (see below).

To identify the 57 loci with a known or predicted role in peripheral nerve myelination we performed the following PubMed searches in September 2014: (1) each gene name plus ‘Schwann’; and (2) each gene name plus ‘Myelin’. We also searched for each gene name plus ‘Schwannoma’ in the GEO Profiles database at NCBI to determine if gene expression is depleted upon treatment with SOX10 siRNA [32].

### Luciferase reporter gene expression constructs

Primers containing attB1 and attB2 Gateway cloning (Invitrogen) sequences were designed for each region identified by our computational pipeline (human gene-specific primer sequences are provided in Additional file 5; rat gene-specific primer sequences are available upon request). Regions were PCR-amplified using human or rat genomic DNA and subsequently cloned into pDONR221 using BP clonase (Invitrogen) according to the manufacturer’s instructions. The pDONR221 constructs were sequence verified and cloned upstream of an E1B minimal promoter [55] directing luciferase expression in the forward (pE1B Forward) or reverse (pE1B Reverse) orientation using LR clonase (Invitrogen) according to the manufacturer’s instructions. Mutagenic primers to delete SOX10 consensus sequences (available upon request) were designed and site directed mutagenesis performed using the QuikChange II Mutagenesis Kit (Agilent Technologies) according to the manufacturer’s instructions. The resulting mutant constructs were sequence verified to ensure that the SOX10 consensus site was deleted and that no other mutations were generated. Subsequently, the constructs were recombined into the pE1B luciferase vector as described above.

### Cell culture and luciferase assays

Cultured rat Schwann cells (S16) [21, 22] were obtained ten years ago from Richard Quarles (NIH/NINDS) and mouse motor neuron derived cells (MN1) [23] were obtained 12 years ago from Kurt Fischbeck (NIH/NINDS). Both cell types were maintained in Dulbecco’s modified Eagle’s medium supplemented with 10 % fetal bovine serum (Gibco) and 2 mM L-glutamine (Gibco). For luciferase assays, 1x10^4^ cells were plated in each well of a 96-well culture plate (Corning Life Sciences) and incubated overnight at 37 °C in 5 % CO_2_. Cells were transfected using Lipofectamine 2000 (Invitrogen) according to the manufacturer’s instructions. Each well was transfected with 200 ng of the pE1B construct [55] and 2 ng of pCMV-Renilla (to correct for experimental variation caused due to cell viability and transfection efficiency) in 50 uL of Opti-MEM (Gibco) and 0.25 uL Lipofectamine 2000. Cells were incubated at 37 °C for 4 h and then grown in standard growth media for 48 h. For overexpression studies, 100 ng of a construct to express wild-type or E189X SOX10 [8], wild-type SOX8, SOX9, or EGR2 was included in the transfection reaction. 48 h after transfection the cells were lysed in 20 uL of 1X passive lysis buffer (Promega) for 1 h. 10 uL of the lysate was transferred to a 96-well assay plate (Corning Life Sciences) and a Dual Luciferase Assay (Promega) was performed to determine the activities of luciferase and renilla. Luciferase activity was normalized to renilla activity and the fold increase in luciferase activity was calculated relative to the empty control vector, which does not contain a genomic insert. For each genomic segment, three independently isolated reporter gene constructs were studied in eight technical replicates for a total of 24 reactions per segment. Statistical calculations comparing the regulatory activity of different alleles were performed using the Mann–Whitney-Wilcoxon test.

### Standard and quantitative RT-PCR

Total RNA was isolated from S16 and MN1 cells. 1x10^5^ cells were plated in a 6-well plate and incubated overnight at 37 °C in 5 % CO_2_. Each well was transfected using 4 ug of wild-type or E189X SOX10 [8] in 1 mL of Opti-MEM and 10 uL of Lipofectamine 2000. Mock transfections were performed in the absence of DNA. Cells were incubated at 37 °C for 4 h and then grown in standard media. After 72 h, total RNA was isolated from the transfected cells using the RNeasy kit (Qiagen). Subsequently, cDNA was synthesized using 1 ug of total RNA and the High Capacity cDNA Reverse Transcription Kit (Life Technologies) according to manufacturer’s instructions. RT-PCR was performed on isolated cDNA using gene specific primers (sequences available upon request). A PCR for *β-actin* served as a positive control. All PCR products were subjected to DNA sequencing to confirm specificity. RNA was purified from three independent rat sciatic nerves at the P1, P15, and adult timepoints using the Qiagen RNeasy Lipid kit, and quantitative RT-PCR was performed as described [20].

### 5’ Rapid amplification of cDNA ends

First strand cDNA libraries were synthesized using total RNA isolated from S16 cells and a primer designed within exon 5 of *Sox6*. The cDNA was TdT-tailed using the 5’RACE System (Invitrogen). Subsequently, two nested PCRs were performed: one using a reverse primer designed within exon 4 of *Sox6* and a second using a reverse primer designed within exon 3 of *Sox6* (primer sequences available upon request). The nested PCR products were separated on a 1 % agarose gel, excised, and purified using the QIAquick gel extraction kit (Qiagen). Gel purified PCR products were TA cloned (Invitrogen) according to manufacturers instructions and 48 clones were subjected to Sanger sequencing: 44 of the resulting sequences correctly mapped to the rat *Sox6* locus.

### siRNA-mediated depletion of SOX10

Control siRNA (siControl 1, Ambion catalog number AM4611) or Sox10 siRNA (siSox10 1, Life Technologies catalog number s131239) were transfected into S16 cells as described using the Amaxa Nucleofection system following the manufacturer’s instructions. At 48 h post-transfection, RNA was isolated using Tri-Reagent (Ambion) and analyzed by quantitative RT-PCR as described [20].

### DNase hypersensitivity site identification

DNase-seq was performed with three biological replicates of rat Schwann (S16) cells at passage numbers five, eight, and 14. Each replicate contained ~20 million cells frozen into 1 ml of freezing media. Cells were thawed and DNase-seq libraries generated as previously described [59] with the exception of adding a 5’ phosphate to linker 1 to increase the ligation efficiency. DNase-seq libraries from three replicates were pooled into one lane of an Illumina Hi-Seq 2000. Raw reads were aligned to the rat (rn5) genome using Bowtie [60] and unique mapping with up to two mismatches allowed in an alignment. For the three samples, 69.2 % (36,295,401), 70.8 % (43,564,606), and 67.9 % (39,579,719) of the reads mapped to rn5. Peaks were called using F-Seq and the default settings [58]. For the three samples: 502,787 (sample 1), 438,254 (sample 2), and 412,267 (sample 3) peaks were identified. 149,342 were shared among all three samples (Additional file 12). We next used sample 2 as a representative experiment and extracted all DNase-seq peaks with an F-Seq score [52] of at least 0.08. This revealed a set of 31,845 peaks (7.3 %; Additional file 13) that were used to prioritize SOX10 response elements (see section ‘Computational identification and prioritization of SOX10 consensus sequences’ above). Data from the three samples have been submitted to GEO (GSM2166058, GSM2166059 and GSM2166060).

## Additional files

Additional file 1: Monomeric SOX10 consensus sequences in the human genome provided as a UCSC Human Genome Browser (hg18) custom track. The fourth column (‘name’) shows the predicted SOX10 binding site sequence. (ZIP 222208 kb)
Additional file 2: Dimeric SOX10 consensus sequences with an intervening sequence of five to 10 base pairs in the human genome provided as a UCSC Human Genome Browser (hg18) custom track. The fourth column (‘name’) shows the predicted SOX10 binding site sequence. (TXT 22732 kb)
Additional file 3: Genomic segments at least five base pairs long and conserved between human, mouse, and chicken provided as a UCSC Human Genome Browser (hg18) custom track. The fourth column (‘name’) shows the position and length of the conserved segment. (TXT 87756 kb)
Additional file 4: Genomic coordinates (hg18; UCSC Human Genome Browser) of 238 SOX10 consensus sequences that are conserved between human, mouse, and chicken and that map to non-coding sequences near a RefSeq gene. Columns are labeled in the file. (XLSX 29 kb)
Additional file 5: Genomic coordinates (hg18; UCSC Human Genome Browser) and primer sequences for 57 genomic segments studied in luciferase assays presented in Fig. 1. Columns are labeled in the file. (XLSX 44 kb)
Additional file 6: Figure S1. Three genomic regions display orientation-specific regulatory activity in motor neurons. The seven active regions from Fig. 1 were tested in forward (grey bars) and reverse (white bars) orientation in mouse motor neuron (MN1) cells. Luciferase data are expressed relative to a control vector without a genomic segment (‘Empty’). The scale of the y-axis is the same as in Fig. 2 to allow comparisons. Error bars indicate standard deviations. (PDF 97 kb)
Additional file 7: Figure S2. SOX8 and SOX9 also increase the regulatory activity of SOX10-CCS-13, SOX10-CCS-19, and SOX10-CCS-51. Luciferase reporter gene constructs harboring SOX10-CCS-13, SOX10-CCS-19, or SOX10-CCS-51 were transfected into mouse motor neurons (MN1) with constructs to express SOX8 or SOX9. The luciferase activity associated with each construct in the presence of SOX8 or SOX9 is expressed relative to that of the same construct in the absence of these transcription factors. Error bars indicate standard deviations. Please note that the SOX10 data are identical to those in Fig. 3a and are included to facilitate a comparison. (PDF 330 kb)
Additional file 8: Figure S3. EGR2 does not act synergistically with SOX10 to activate SOX10-CCS-13, SOX10-CCS-19, or SOX10-CCS-51 in vitro. Luciferase reporter gene constructs harboring SOX10-CCS-13, SOX10-CCS-19, or SOX10-CCS-51 were transfected into mouse motor neurons (MN1) with constructs to express EGR2 and/or SOX10. The luciferase activity associated with each construct in the presence of the transcription factor(s) is expressed relative to that of the untreated reporter construct. Error bars indicate standard deviations. Please note that the SOX10 data are identical to those in Fig. 3a and are included to facilitate a comparison. (PDF 328 kb)
Additional file 9: Genomic coordinates and custom tracks (rn5; UCSC Rat Genome Browser) for SOX10 ChIP-seq peaks that map to both a DNase hypersensitivity site and at least one SOX10 monomeric consensus sequence that is conserved between human, mouse, and chicken. Columns are labeled in the file. (XLSX 23 kb)
Additional file 10: Figure S4. SOX10 regulates genes that inhibit glial cell differentiation in vivo. Primary Schwann cells were extracted and grown from three independent rat adult sciatic nerves. Cells were treated with a control siRNA or an siRNA targeted against *Sox10* as in Fig. 7c. Quantitative RT-PCR was used to measure expression levels of each indicated gene. The effect on expression of each gene (indicated across the bottom) is expressed relative to the control siRNA and error bars indicate standard deviations. Please note that, consistent with the in vitro data, *Hmga2* did not show a decrease in expression levels in vivo (data not shown). (PDF 334 kb)
Additional file 11: SOX10 ChIP-seq peaks in the rat genome using HOMER analysis. The data are provided as a UCSC Rat Genome Browser (rn5) custom track. (TXT 1526 kb)
Additional file 12: DNase-seq peaks identified in S16 cells that are common to all three experimental replicates. The data are provided as a UCSC Rat Genome Browser (rn5) custom track and the fourth column (‘name’) shows the F-Seq score for the peak. (TXT 5171 kb)
Additional file 13: DNase-seq peaks identified in S16 cells from experimental sample 2 that have an F-Seq score of at least 0.08. The data are provided as a UCSC Rat Genome Browser (rn5) custom track and the fourth column (‘name’) shows the F-Seq score for the peak. (TXT 1075 kb)

## Abbreviations

CCS: Conserved Consensus Sequence
ChIP-seq: Chromatin immunoprecipitation followed by sequencing
DNase-seq: DNase I hypersensitivity sites followed by sequencing
MCS: Multiple species Conserved Sequence
PCR: Polymerase Chain Reaction
PNS: Peripheral Nervous System
SOX10: Sry-box 10
