# Supplementary figures and images for: Stringent comparative sequence analysis reveals SOX10 as a putative inhibitor of glial cell differentiation
Source: BMC Genomics. 2016 Nov 7;17:887. doi: 10.1186/s12864-016-3167-3 (PMC5100263; doi:10.1186/s12864-016-3167-3)

# Motor Neurons

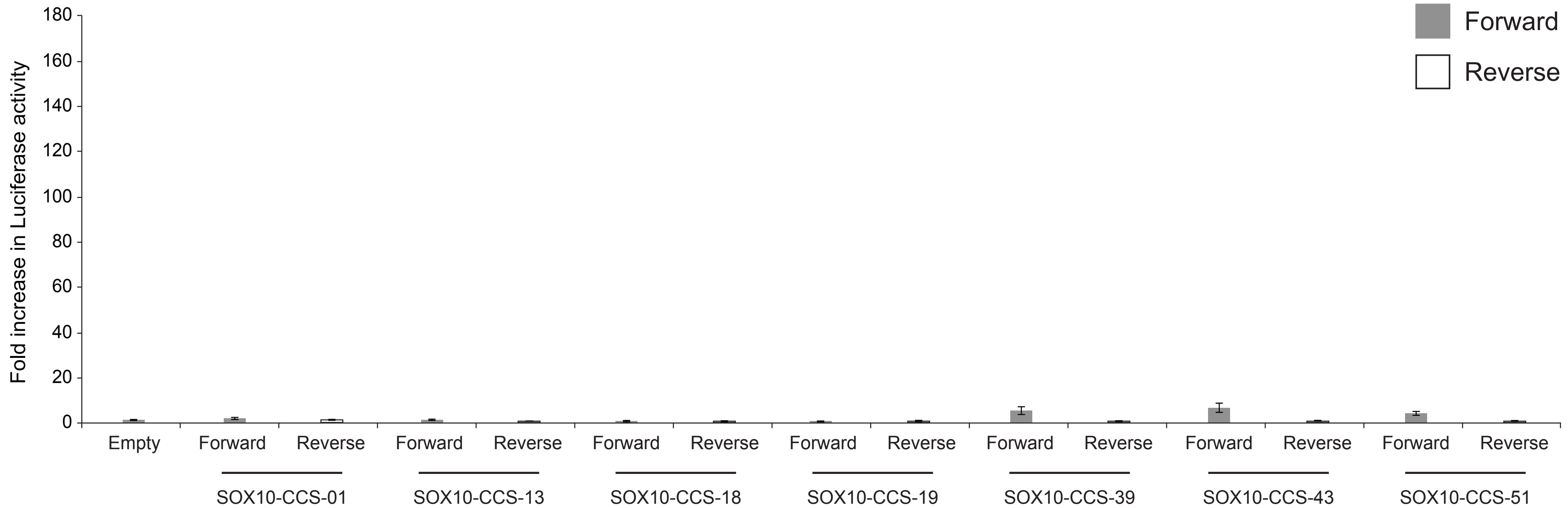

Supplement: Additional file 6: Figure S1. — Three genomic regions display orientation-specific regulatory activity in motor neurons. The seven active regions from Fig. 1 were tested in forward (grey bars) and reverse (white bars) orientation in mouse motor neuron (MN1) cells. Luciferase data are expressed relative to a control vector without a genomic segment (‘Empty’). The scale of the y-axis is the same as in Fig. 2 to allow comparisons. Error bars indicate standard deviations. (PDF 97 kb) [file 12864_2016_3167_MOESM6_ESM.pdf]

# Motor Neurons (MN1)

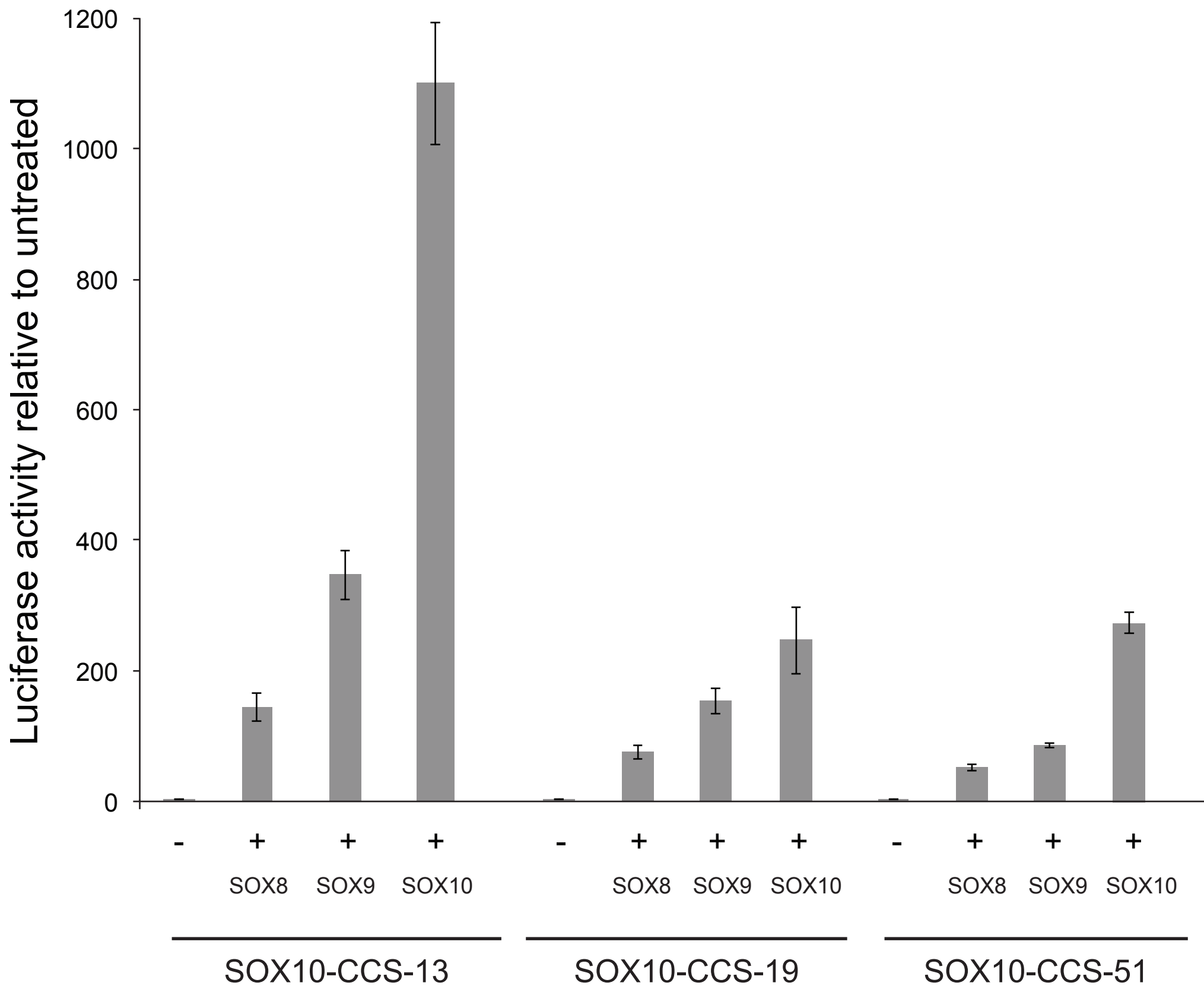

Supplement: Additional file 7: Figure S2. — SOX8 and SOX9 also increase the regulatory activity of SOX10-CCS-13, SOX10-CCS-19, and SOX10-CCS-51. Luciferase reporter gene constructs harboring SOX10-CCS-13, SOX10-CCS-19, or SOX10-CCS-51 were transfected into mouse motor neurons (MN1) with constructs to express SOX8 or SOX9. The luciferase activity associated with each construct in the presence of SOX8 or SOX9 is expressed relative to that of the same construct in the absence of these transcription factors. Error bars indicate standard deviations. Please note that the SOX10 data are identical to those in Fig. 3a and are included to facilitate a comparison. (PDF 330 kb) [file 12864_2016_3167_MOESM7_ESM.pdf]

Motor Neurons (MN1)

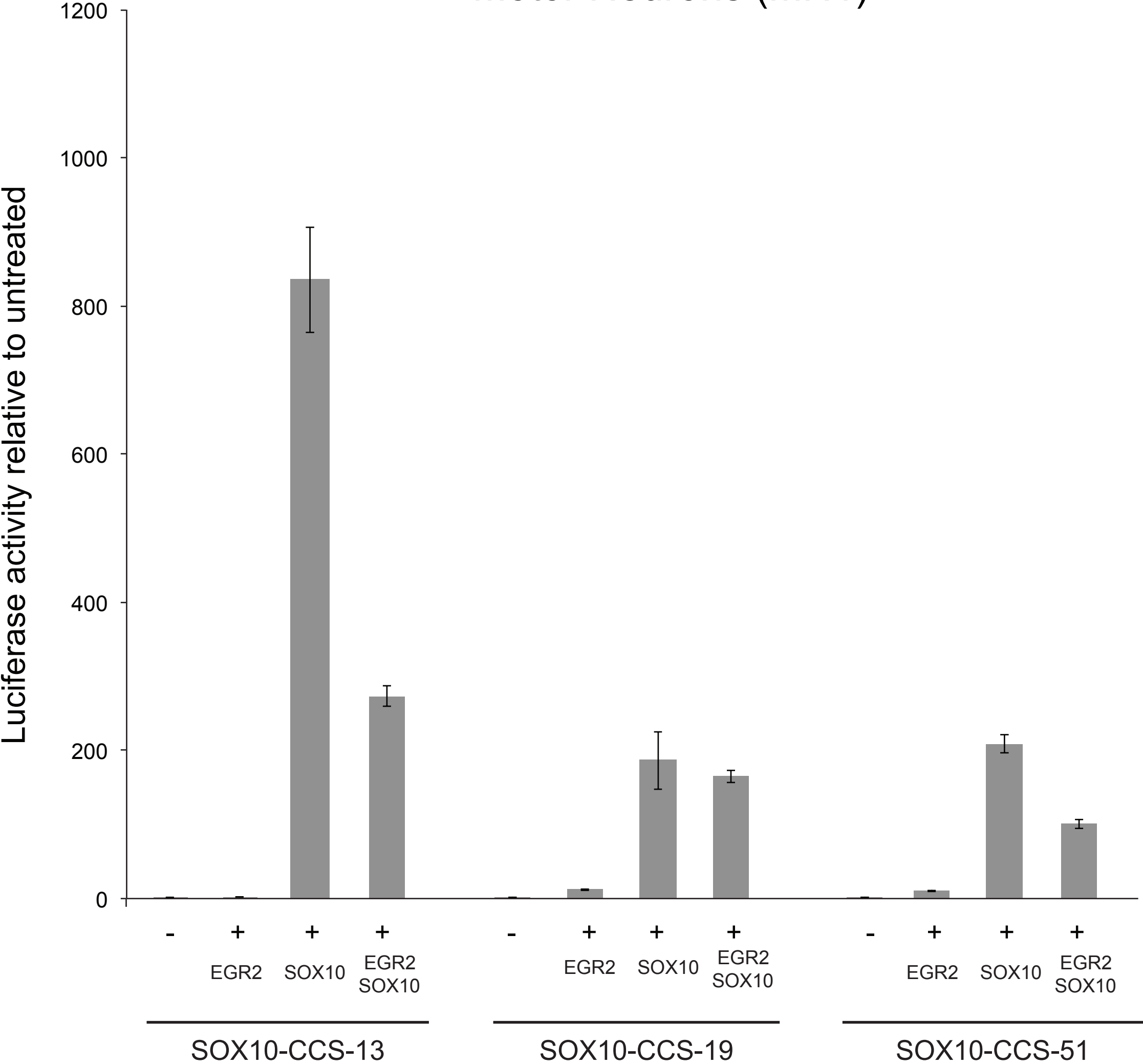

Supplement: Additional file 8: Figure S3. — EGR2 does not act synergistically with SOX10 to activate SOX10-CCS-13, SOX10-CCS-19, or SOX10-CCS-51 in vitro. Luciferase reporter gene constructs harboring SOX10-CCS-13, SOX10-CCS-19, or SOX10-CCS-51 were transfected into mouse motor neurons (MN1) with constructs to express EGR2 and/or SOX10. The luciferase activity associated with each construct in the presence of the transcription factor(s) is expressed relative to that of the untreated reporter construct. Error bars indicate standard deviations. Please note that the SOX10 data are identical to those in Fig. 3a and are included to facilitate a comparison. (PDF 328 kb) [file 12864_2016_3167_MOESM8_ESM.pdf]

# siSox10 in primary Schwann cells

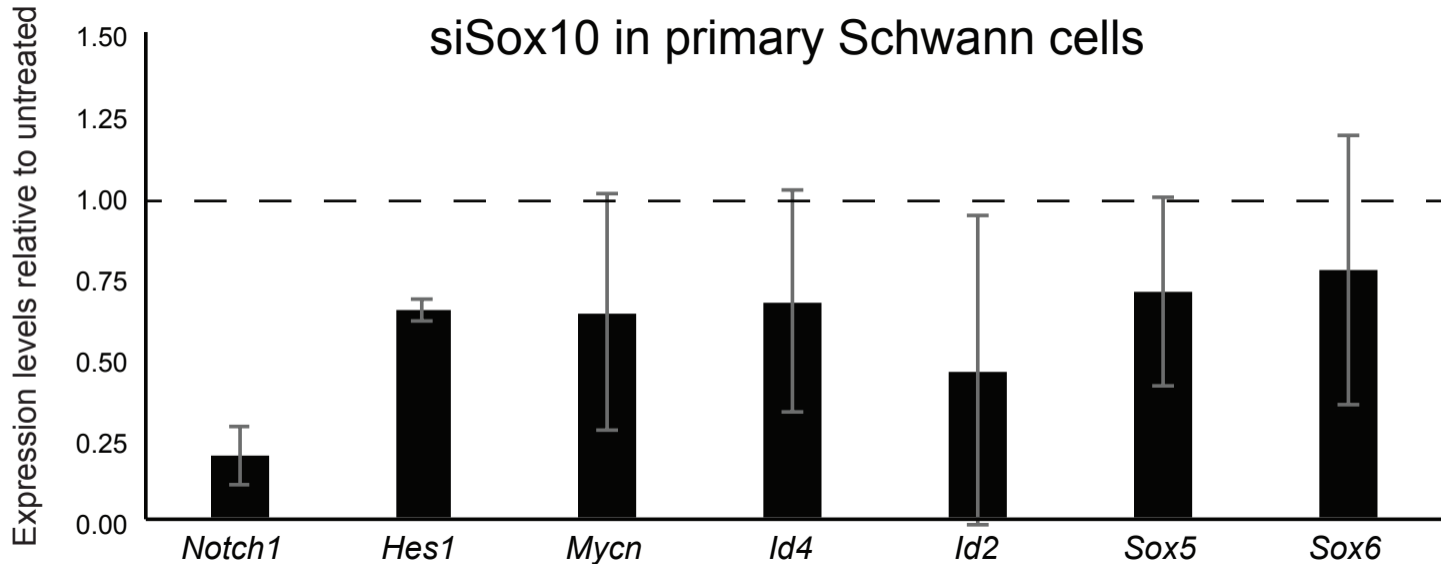

Supplement: Additional file 10: Figure S4. — SOX10 regulates genes that inhibit glial cell differentiation in vivo. Primary Schwann cells were extracted and grown from three independent rat adult sciatic nerves. Cells were treated with a control siRNA or an siRNA targeted against Sox10 as in Fig. 7c. Quantitative RT-PCR was used to measure expression levels of each indicated gene. The effect on expression of each gene (indicated across the bottom) is expressed relative to the control siRNA and error bars indicate standard deviations. Please note that, consistent with the in vitro data, Hmga2 did not show a decrease in expression levels in vivo (data not shown). (PDF 334 kb) [file 12864_2016_3167_MOESM10_ESM.pdf]
